# Efficacy of Periodontal Surgery on Subgingival Periodontopathogen Levels in Periodontitis Patients: A Randomized Clinical Trial

**DOI:** 10.1155/ijod/4419048

**Published:** 2025-12-26

**Authors:** Cansu Bayram, Ece Açıkgöz-Alparslan

**Affiliations:** ^1^ Department of Periodontology, Tekirdağ Oral and Dental Health Hospital, Tekirdağ, Türkiye; ^2^ Department of Periodontology, Faculty of Dentistry, Trakya University, Edirne, Türkiye, trakya.edu.tr

**Keywords:** periodontitis, *Porphyromonas gingivalis*, real-time PCR, *Tannerella forsythia*, *Treponema denticola*

## Abstract

**Objectives:**

This study evaluates the changes in subgingival periodontopathogens following access flap surgery (AFS) in Stage III Grade B periodontitis patients.

**Materials and Methods:**

This randomized clinical split‐mouth study included 20 systemically healthy periodontitis patients. Participants’ right and left quadrants were randomly assigned to the control (nonsurgery) or test (AFS) group. Quantities of subgingival periodontopathogens (*Porphyromonas gingivalis*, *Treponema denticola*, and *Tannerella forsythia*) and periodontal measurements (gingival index [GI], plaque index [PI], bleeding on probing [BOP], probing depth [PD], and clinical attachment level [CAL]) were evaluated before and 4 weeks after surgery. Quantitative polymerase chain reaction was utilized for detecting and quantifying periodontopathogens.

**Results:**

A significant reduction in periodontopathogens was observed in the test group, while levels increased in the control group (*p*  < 0.05). A significant difference was noted between the groups in changes in the quantities of periodontopathogens (*p*  < 0.05). The reduction in periodontal parameters was significantly greater in the test group 1‐month post‐AFS (*p*  < 0.05). Positive correlations were observed between decreases in *P. gingivalis* levels and reductions in *T. denticola* and *T. forsythia* (*p*  < 0.05).

**Conclusions:**

AFS effectively reduces subgingival periodontopathogens and enhances periodontal health in the early healing phase. It demonstrates significant benefits for patients with advanced periodontitis and deep residual pockets.

**Clinical Relevance:**

AFS is an effective treatment option for managing deep residual pockets, as it promotes periodontal health and significantly reduces bacterial burden during the critical early healing phase. These results are particularly valuable for clinicians aiming to prevent disease progression, thereby enhancing patient care and treatment outcomes.

**Trial Registration:** ClinicalTrials.gov identifier: NCT06684288

## 1. Introduction

Periodontal disease is characterized by an inflammatory process arising from interactions between pathogenic biofilm and the host response [1]. Notably, pathogens from the red complex (*Porphyromonas gingivalis*, *Treponema denticola*, and *Tannerella forsythia*) are closely associated with periodontal disease [2] and pockets exceeding 6 mm in depth [3]. The interactions among red‐complex pathogens lead to substantial changes in both the composition and metabolic activities of the biofilm [4]. In the keystone plaque hypothesis, Hajishengallis et al. [5] demonstrated that *P. gingivalis* can induce periodontitis even at low densities by significantly altering bacterial organization. When the balance between the host inflammatory response and periodontopathogens is disrupted, microbial dysbiosis occurs, leading to irreversible loss of periodontal attachment and alveolar bone [4].

The ultimate goal of periodontal treatment is to reduce the risk of tooth loss, decrease the level of periodontopathogens, and prevent further disease progression [6]. Periodontitis treatment should adopt a stepwise approach, beginning with thorough oral hygiene instructions and risk factor management. Subgingival instrumentation (SI), a key component of the second step of treatment, is crucial for establishing and maintaining periodontal health [7]. Nevertheless, the efficacy of SI may be constrained by challenges in thoroughly instrumenting deep periodontal pockets, frequently resulting in incomplete microbial elimination [8]. To enhance prognosis, the third step (the surgical phase) is often considered when periodontal treatment goals are not achieved in the second step, particularly when deep pockets (≥6 mm) persist [6]. This step aims to surgically address deep pockets, achieve complete instrumentation, and ensure thorough removal of pathogenic subgingival biofilm [7]. Recent data suggest that surgical treatment of deep residual pockets has the potential to induce a microbiological shift more conducive to periodontal health [6]. Access flap surgery (AFS) comprises a variety of surgical techniques designed to access the root surface, and papilla preservation approaches are identified as a subgroup of AFS and do not involve the active removal of alveolar bone and soft tissues. Recently, using a microsurgical approach to elevate, handle, and suture access flaps has been shown to improve the percentage of primary closure achieved during the healing process [6, 9].

The limited evidence concerning alterations in the quantities of disease‐associated pathogenic species, particularly following microsurgical AFS, necessitates further research. The objective of the present randomized controlled trial is to determine the effect of AFS on the short‐term changes in the quantities of *P. gingivalis* (*Pg*), *T. denticola* (*Td*), and *T. forsythia* (*Tf*).

## 2. Methods

In this split‐mouth, randomized controlled clinical trial, 20 individuals (40 cases: 20 controls and 20 test subjects) participated, all of whom were comprehensively informed about the study’s objectives and provided their written consent. The protocol of this clinical research was approved with the EKAEK 05/07 code by the Clinical Research Ethics Committee of Trakya University. The registration of the study protocol in ClinicalTrials.gov followed the ethical principles of the Declaration of Helsinki and the CONSORT guidelines.

### 2.1. Study Population

Individuals diagnosed with stage III grade B periodontitis, according to the criteria established at the 2017 World Workshop on the Classification of Periodontal Diseases and Conditions, were included in the study [10]. Inclusion criteria were defined as follows: having (i) 20 or more permanent teeth, excluding third molars and teeth with endodontic lesions; (ii) periodontitis with > 30% loss of periodontal support; (iii) at least two nonadjacent teeth with probing depth (PD) ≥ 6 mm, clinical attachment level (CAL) ≥ 5 mm, and radiographic bone loss extending to at least middle of the root [11]; (iv) bleeding on probing (BOP) in ≥ 40% of periodontal sites [12]; and (v) at least one tooth per quadrant meeting the PD and CAL criteria. Periodontitis severity was assessed by calculating the percentage of bone loss relative to age, yielding values between 0.25 and 1.

Exclusion criteria were (i) pregnancy or lactation; (ii) systemic diseases affecting immune response (e.g., diabetes, bone metabolic diseases, HIV, immunosuppressive therapy, and radiation); (iii) caries, restorations, or prostheses near the sampling site; and (iv) use of anti‐inflammatory drugs or antibiotics in the last 3 months.

NSPT was administered to patients scheduled for participation in the study, using periodontal hand instruments (Gracey Curettes, Hu‐Friedy; Chicago, IL) in conjunction with an ultrasonic device (Cavitron Plus, Dentsply, Duisburg, Germany). Each patient underwent the first step of treatment, which involved the mechanical removal of supragingival deposits, motivation, and personalized oral hygiene instructions, including interdental plaque control and toothbrushing using the modified Bass technique [7]. In the second step, SI was completed on all four quadrants in a single appointment by a blinded periodontist (C.B.) within the first week. One month after SI (14 days before surgery), patients were clinically re‐examined, and inclusion/exclusion criteria were reassessed. This interval was chosen in accordance with the EFP S3 clinical practice guideline [7], which recommends reassessment after an adequate healing period. Previous evidence indicates that the early healing phase and initial reductions in inflammation can usually be detected within the first month, while recolonization of periodontopathogens may also occur during this period [13, 14]. Therefore, the 4‐week interval was considered a clinically relevant time point for re‐evaluation prior to surgical intervention.

Patients with deep residual pockets (PD ≥ 6 mm) were selected for AFS and underwent randomization, with one side receiving AFS (test group) and the other left untreated (control group). Patients who did not require AFS were excluded and instead enrolled in repeated SI or supportive periodontal care [7].

### 2.2. Outcomes

The primary outcome was the mean fold change in the quantities of target pathogens (*P. gingivalis*, *T. denticola*, and *T. forsythia*) at the 1‐month follow‐up in Stage III Grade B periodontitis patients following AFS. The secondary outcomes included the percentage of sites with positive BOP, as well as the mean values of PI, GI, PD, and CAL.

### 2.3. Randomization and Allocation Concealment

Patients were assigned consecutive numbers during enrollment, and treatment quadrants were randomized using a computer‐generated sequence by an investigator not involved in the treatment. Allocation concealment was ensured with sequentially numbered, opaque sealed envelopes. The right or left side was designated as the test group, and the opposite side as the control group. The allocation remained blinded to the examiner (E.A.A.) during the periodontal examination, and the operator (C.B.) opened the envelope only at the first treatment visit.

### 2.4. Treatment Procedure

Each patient who met the study criteria underwent one of the following: One side of the mouth was treated with AFS, while the contralateral quadrants served as the control. All the access surgeries were conducted by the same periodontist (C.B.) who was blinded to periodontal parameters and microbiological sampling sites. A mucoperiosteal flap was elevated and handled with microsurgical instruments (blades, scalpel, needle holder, scissor, and tweezer, Carl Martin GmbH, Solingen, Germany) in accordance with the simplified papilla preservation flap (SPFF) technique [9, 15]. Granulation tissue attached to the alveolar bone was excised to ensure complete access and optimal visibility of the root surfaces. Instrumentation was performed using a combination of ultrasonic and hand instruments, with no alveolar bone modification. Subsequently, the mucoperiosteal flap was repositioned and primarily closed with proximal simple sutures employing monofilament suture material (5/0 Propilen, Dogsan, Türkiye). Patients were given postoperative recommendations; however, no medication was prescribed.

In the present study, control sites were not subjected to additional retreatment after the initial SI. This approach was selected to maintain the control sites as a valid comparator for the surgically treated quadrants and to eliminate potential confounding effects that could arise from repeated instrumentation.

### 2.5. Clinical Periodontal Evaluations

Periodontal measurements including PD (mm), CAL (mm), BOP (±), GI, and PI [16] were assessed at two distinct time intervals (prior to and 1 month after AFS) by a calibrated periodontist (E.A.A.), who was blinded to the experimental sites [17]. These measurements were conducted on six different aspects of each tooth (disto‐buccal/labial, midbuccal/labial, mesio‐buccal/labial, disto‐lingual/palatinal, midlingual/palatinal, and mesio‐lingual/palatinal) using a manual periodontal probe (Williams Probe, Hu‐Friedy; Chicago, IL), and the average value was computed.

### 2.6. Collection of Subgingival Plaque Samples

Among the periodontal scores recorded for each patient, four teeth (two for the treatment group and two for the control group) demonstrating the highest scores (PD ≥ 6 mm, CAL ≥ 5 mm) were designated as donor sites for subgingival plaque sampling. Sample collection was conducted before and 1 month after surgery by the periodontist (E.A.A.) responsible for performing clinical measurements. Sampling sites were isolated using sterile cotton rolls, followed by the supragingival plaque removal from the tooth surface. Sterilized paper points (Paperpoint #50, EndoPoint, Paraiba do Sul, RJ, Brazil) were then delicately inserted into the periodontal pockets, applying gentle pressure until mild resistance was felt [18]. They were held for 20 s and then transferred to sterile tubes for storage at −80°C.

### 2.7. Microbiological Analyses

Molecular genetic identification and quantification of subgingival periodontopathogens were performed by a blinded operator using DNA isolated with the PureLink Genomic DNA Mini Kit (Invitrogen, Massachusetts, USA) following the manufacturer’s instructions. The isolated DNA samples were stored at −20°C until qPCR analysis.

Real‐time qPCR was performed using TaqMan Probes (Applied Biosystems, CA, USA), labeled with fluorochromes at the 5′ and 3′ termini, and the TaqMan Multiplex Master Kit, which contains all necessary components. Reference primers and probe sequences for the bacterial strains used were based on Coffey et al.’s [19] study and are listed in Table 1. The samples were run on an ABI 7500 Fast Real‐Time PCR System (Applied Biosystems, CA, USA). The qPCR protocol included a polymerase activation step (1 cycle at 95°C for 3 min), a denaturation step (40 cycles at 95°C for 15 s), and an annealing/extension step (40 cycles at 60°C for 30 s). The threshold cycle (Ct) values obtained from the analysis of subgingival samples were incorporated into the 2^−*Δ*Ct^ formula to calculate the fold difference [20].

**Table 1:** TaqMan oligos and TaqMan probes used in the real‐time assays.

Target bacteria	Description	Primer sequences (5′→3′)	Amplicon size
*Porphyromonas gingivalis*	TaqMan forward oligo	CTGCGTATCCGACATATC	134 bp
	TaqMan reverse oligo	GGTACTGGTTCACTATCG	—
	TaqMan Prob	ABY‐ACCATAGACGACGGAGCACC‐QSY	—

*Treponema denticola*	TaqMan forward oligo	GTTGTTCGGAATTATTGG	109 bp
	TaqMan reverse oligo	GATTCAAGTCAAGCAGTA	—
	TaqMan Prob	FAM‐TCACACCAGGCTTACC‐QSY	—

*Tannerella forsythia*	TaqMan forward oligo	GAGGTTGTGGAAGGTATG	108 bp
	TaqMan reverse oligo	GTAGATCAGAATGTACGGATT	—
	TaqMan Prob	VIC‐TCTCCGCTTATTTCGTGAC‐QSY	—

The disparity between the target bacteria detected at baseline and after 1 month was calculated using the following formulas:

ΔCt:Cttarget bacteria in1st month−Cttarget bacteria in baseline

2−Δ∆Ct: The fold change in quantities of the target bacteria on 1st month compared to baseline.

### 2.8. Statistical Analysis

All analyses were conducted using the SPSS version 23 software package (SPSS Inc., Chicago, IL, USA). Two study groups were defined based on a substantial effect size (0.4) difference in microbial quantities, with a significance level of 0.05 and a power of 80% [21]. To assess correlations in repeated measurements with a correlation coefficient of 0.5, a sample size of 40 cases (20 test–20 control) was determined using ${\text{G}}^{{,}^{*}}$Power 3.1.9.2 software.

The intraobserver agreement, assessed through reliability analysis of the investigator’s measurements, was evaluated in five periodontitis patients not included in this study. The intraclass correlation coefficient (ICC) and confidence intervals (CI) were calculated by repeating the CAL measurements for each tooth at a 48‐h interval.

Continuous variables were tested for normality using the Shapiro–Wilk test. Depending on the distribution, comparisons between independent groups were made using the Student’s *t*‐test or Mann–Whitney *U* test and between dependent groups using the Paired *t*‐test or Wilcoxon test. Spearman correlation analysis was used to assess relationships between continuous variables. Continuous variables are presented as mean ± standard deviation (SD), median (IQR), and range (minimum–maximum), while categorical variables are presented as frequencies and percentages.

## 3. Results

The intraobserver agreement analysis yielded a satisfactory ICC (*p*  < 0.001). The ICC coefficient for intraobserver agreement was 0.989 (95% CI: 0.98–0.99; *p*  < 0.001).

**Figure 1:**
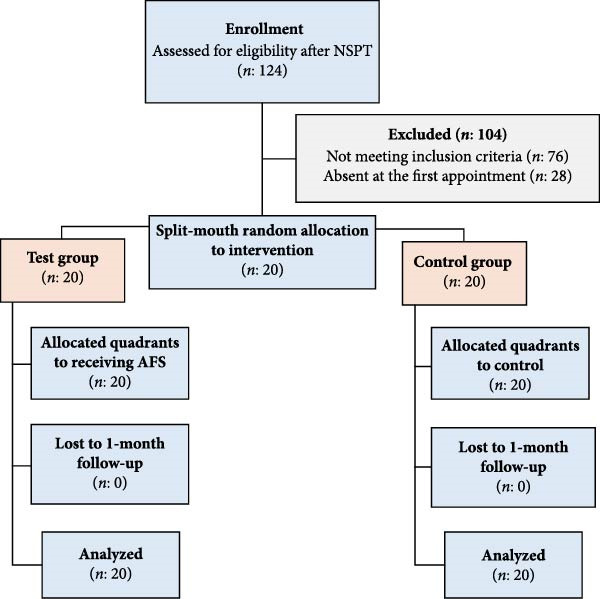
Study design flowchart.

Figure 1 illustrates the study design flowchart. A total of 20 individuals participated in the study, comprising 11 males (55%) and nine females (45%), and age range varied between 36 and 58 years, with a mean age of 46.3 ± 5.8 years.

### 3.1. Microbial Assessment

**Figure 2:**
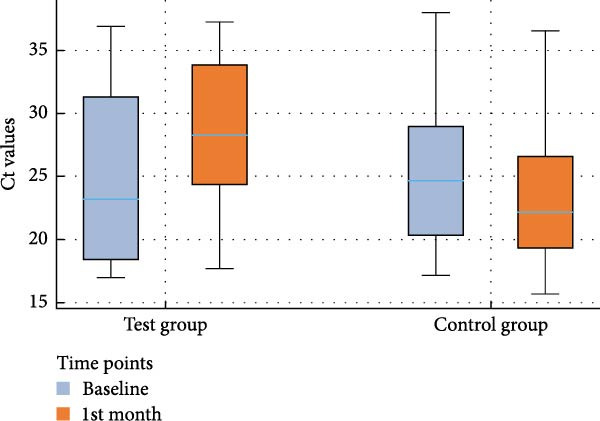
Box‐and‐whisker plot showing Ct values of *P. gingivalis* at baseline and 1st month in the test and control groups.

**Figure 3:**
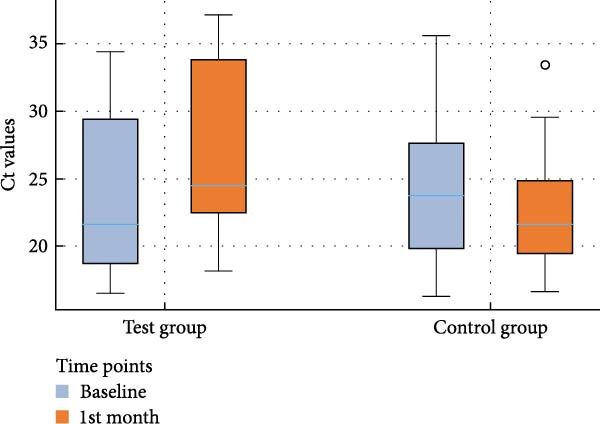
Box‐and‐whisker plot showing Ct values of *T. forsythia* at baseline and 1st month in the test and control groups.

**Figure 4:**
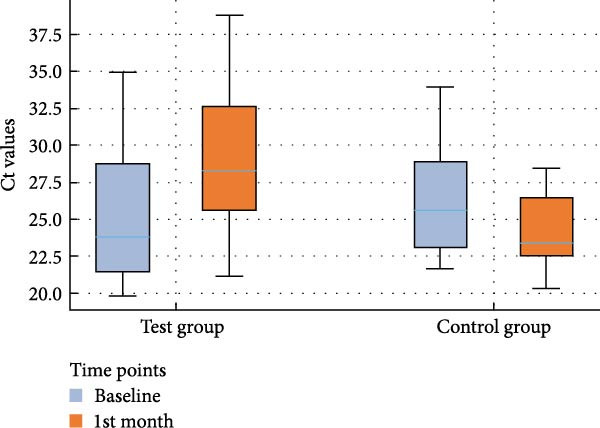
Box‐and‐whisker plot showing Ct values of *T. denticola* at baseline and 1st month in the test and control groups.

The analyses of the Ct values of *Pg*, *Td*, and *Tf* at two distinct time points within both the control and test groups, along with the fold changes in bacterial counts following AFS, are delineated in Table 2. The corresponding distribution of Ct values for each species and study group at baseline and 1 month is depicted in box‐and‐whisker plots (Figures 2–4), providing a visual representation of data dispersion and outliers.

**Table 2:** Baseline and 1st month threshold values of *P. gingivalis*, *T. denticola*, and *T. forsythia*, along with the fold change occurring in each target pathogen between the two time points .

Bacterial species	Baseline (*C*_t_)	1st month (*C*_t_)	*t*	*p*_2_	Fold change in bacterial counts (2^*−*∆∆Ct^)
*P. gingivalis*					
Control					
Mean ± SD	25.89 ± 6.2	23.37 ± 5.7	3.722	**0.001** *⁣* ^∗∗^	48.55 ± 112.6
Median (IQR)	24.75 (9)	22.17 (7.9)			3.35 (26.2)
Min–max	17.09–37,93	15.67–36.51			−2.44–469.83
Test					
Mean ± SD	24.8 ± 6.8	28.63 ± 6.3	−6.583	**<0.001** *⁣* ^∗∗∗^	−60.53 ± 120.1
Median (IQR)	23.17 (13.2)	28.53 (10.4)			−12.21 (65.9)
Min–max	16.95–36.9	17.64–37.25			−509.8–1.17
*t*/*Z*	−0.839	−2.782			7.116
p_1_	0.402^b^	**0.008** *⁣* ^∗∗^^a^			**<0.001** *⁣* ^∗∗∗^^a^
*T. denticola*					
Control					
Mean ± SD	24.28 ± 5.3	22.52 ± 4.2	2.593	**0.018** *⁣* ^∗^	29.47 ± 106.1
Median (IQR)	23.77 (8.6)	21.85 (6.1)			5.01 (12.7)
Min–max	16.32–35.64	16.61–33.42			−9.46–477.85
Test					
Mean ± SD	23.49 ± 5.8	27.35 ± 6.6	−6.788	**<0.001** *⁣* ^∗∗∗^	−46.88 ± 60.8
Median (IQR)	22 (11)	24.36 (12.7)			−12.08 (85.7)
Min–max	16.49–34.37	18.21–37.14			−212.82–0.93
*t*/*Z*	−0.460	−2.326			6.346
p_1_	0.646^b^	**0.020** *⁣* ^∗^^b^			**<0.001** *⁣* ^∗∗^^a^
*T. forsythia*					
Control					
Mean ± SD	26.08 ± 3.5	24.31 ± 2.5	2.954	**0.008** *⁣* ^∗∗^	11.55 ± 20.3
Median (IQR)	25.6 (6.3)	23.45 (4.2)			3.02 (18.7)
Min–max	21.62–33.94	20.26–28.49			−7.04–63.78
Test					
Mean ± SD	24.98 ± 4.3	29.18 ± 4.7	−6.025	**<0.001** *⁣* ^∗∗∗^	−123.46 ± 253.8
Median (IQR)	23.73 (7.7)	28.64 (7.2)			−18.94 (76)
Min–max	19.81–34.95	21.12–38.84			−914.92–1.03
*t*/*Z*	0.883	−4.071			6.497
p_1_	0.383^a^	**<0.001** *⁣* ^∗∗∗^^a^			**<0.001** *⁣* ^∗∗∗^^a^

*Note:* Bold *p*‐values indicate statistically significant differences. p_1_: Comparison between groups (test and control) (^a^Student’s *t*‐test, ^b^Mann–Whitney *U* test); p_2_: Comparison within groups (Baseline and 1st month) (Wilcoxon test). 2^−∆∆Ct^, the fold change in quantities of the target pathogen in the first month compared to the baseline; Ct, threshold cycle values for the target pathogen.

Abbreviations: IQR, interquartile range; SD, standard deviation.

*⁣* ^∗^ *p*  < 0.05.

*⁣* ^∗∗^ *p*  < 0.01.

*⁣* ^∗∗∗^ *p*  < 0.001.

No significant differences were observed in the baseline threshold cycle values for any bacterial species between the groups (*p* > 0.05). However, significant differences were detected for all species (*Pg*, *Td*, and *Tf*) in the 1st‐month values (*p* = 0.008, 0.020, and <0.001, respectively, Table 2). In the test group, a highly significant reduction in the baseline Ct values of each bacterial species was observed within the first month following AFS (*p*  < 0.001 for all species, Table 2). This significant decrease was 12.21‐fold in *Pg*, 12.08‐fold in *Td*, and 18.94‐fold in *Tf*. Nevertheless, the bacterial quantities increased by 3.35‐fold for *Pg*, 5.01‐fold for *Td*, and 3.02‐fold for *Tf* in the control group relative to baseline levels (*p*  < 0.05 for all species, Table 2). Additionally, there was a significant difference between the test and control groups in terms of fold changes in bacterial counts (*2* ^−∆∆*Ct*^) observed in the first month after the AFS (*p*  < 0.001 for all species, Table 2).

### 3.2. Evaluation of Periodontal Parameters

The descriptive statistics for the periodontal parameters recorded at two different time points in the full‐mouth and treatment sites are presented in Table 3. No significant difference was found between the baseline parameters of the control and test groups (*p* > 0.05). However, 1 month post‐treatment, the test group showed significantly lower values for PD, CAL, PI, GI, and BOP compared to the control group (*p* = 0.029, 0.038, 0.015, 0.011, and <0.001, respectively). All periodontal parameters measured at baseline demonstrated a significant decrease in the first month (*p*  < 0.05, Table 3).

**Table 3:** Comparison of baseline and 1‐month periodontal parameters.

Clinical parameters	Full mouth		*p*‐Value	Test site		*p*‐Value
	Baseline	1st month		Baseline	1st month	
PD (mm)						
Mean ± SD	3.62 ± (0.6)	3.47 ± (0.7)	**0.015** *⁣* ^∗^^a^	7.5 ± (1.1)	6.13 ± (1.1)	‬‬‬**<0.001** *⁣* ^∗∗∗^^a^‬‬‬‬‬‬‬‬‬‬‬‬‬‬‬‬‬‬‬‬‬‬‬‬‬‬‬‬‬‬‬‬‬‬‬‬‬‬‬‬‬‬‬‬‬‬‬‬‬‬‬‬‬‬‬‬‬‬‬‬‬‬‬‬‬‬
Median (IQR)	3.63 (1.1)	3.68 (1.1)		7.55 (1)	6.5 (1.6)	
Min–max	2.61–4.6	2.23–4.29		5.6–10	4.3–9	
CAL (mm)						
Mean ± SD	3.74 ± (0.7)	3.6 ± (0.8)	**<0.003** *⁣* ^∗∗^^a^	8.47 ± (1.6)	7.17 ± (1.6)	‬‬‬**<0.001** *⁣* ^∗∗∗^^a^‬‬‬‬‬‬‬‬‬‬‬‬‬‬‬‬‬‬‬‬‬‬‬‬‬‬‬‬‬‬‬‬‬‬‬‬‬‬‬‬‬‬‬‬‬‬‬‬‬‬‬‬‬‬‬‬‬‬‬‬‬‬‬‬‬‬‬
Median (IQR)	3.69 (1.2)	3.72 (1.4)		8 (2.3)	7 (1.9)	
Min–max	2.72–5.24	2.3–4.98		5.6–12	4.3–11	
PI						
Mean ± SD	1.46 ± (0.5)	0.57 ± (0.5)	**<0.001** *⁣* ^∗∗∗^^b^	1.85 ± (0.4)	0.2 ± (0.4)	‬‬‬**<0.001** *⁣* ^∗∗∗^^b^ ‬‬‬‬‬‬‬‬‬‬‬‬‬‬‬‬‬‬‬‬‬‬‬‬‬‬‬‬‬‬‬‬‬‬‬‬‬‬‬‬‬‬‬‬‬‬‬‬‬‬‬‬‬‬‬‬‬‬‬‬‬‬‬‬‬‬
Median (IQR)	1.26 (1)	1 (1)		2 (0)	0 (0)	
Min–max	1–2.28	0–1.28		1–2	0–1	
GI						
Mean ± SD	1.59 ± (0.3)	1.31 ± (0.3)	**<0.001** *⁣* ^∗∗∗^^b^	1.96 ± (0.2)	1.14 ± (0.3)	‬‬‬**<0.001** *⁣* ^∗∗∗^^b^ ‬‬‬‬‬‬‬‬‬‬‬‬‬‬‬‬‬‬‬‬‬‬‬‬‬‬‬‬‬‬‬‬‬‬‬‬‬‬‬‬‬‬‬‬‬‬‬‬‬‬‬‬‬‬‬‬‬‬‬‬‬‬‬‬‬‬‬
Median (IQR)	1.46 (0.6)	1.12 (0.5)		2 (0)	1 (0.2)	
Min–max	1.22–2.18	1.04–2.14		1.25–2	1–2	
BOP (%)						
Mean ± SD	53.54 ± (25.8)	19.12 ± (13.3)	**<0.001** *⁣* ^∗∗∗^^b^ ‬	903 ± (17.2)	28.4 ± (20.5)	‬‬‬**<0.001** *⁣* ^∗∗∗^^a**‬**^ ‬‬‬‬‬‬‬‬‬‬‬‬‬‬‬‬‬‬‬‬‬‬‬‬‬‬‬‬‬‬‬‬‬‬‬‬‬‬‬‬‬‬‬‬‬‬‬‬‬‬‬‬‬‬‬‬‬‬‬‬‬‬‬‬‬‬‬
Median (IQR)	48 (29.6)	14.5 (14.1)		100 (22.5)	28.5 (33.3)	
Min–max	21–100	4–57		46–100	0–75	

*Note:* Bold *p*‐values indicate statistically significant differences between baseline and 1‐month.

Abbreviations: BOP, bleeding on probing; CAL, clinical attachment level; GI, gingival index; IQR, interquartile range; PD, probing depth; PI, plaque index; SD, standard deviation.

^a^Paired samples *t* test.

^b^Wilcoxon test.

*⁣* ^∗^ *p*  < 0.05.

*⁣* ^∗∗^ *p*  < 0.01.

*⁣* ^∗∗∗^ *p*  < 0.001.

Table 4 details the changes in periodontal parameters for teeth from which plaque samples were obtained, comparing the first month to baseline. The decrease in each parameter (PD, CAL, PI, GI, and BOP) was significantly greater in the test group (*p* = 0.001, <0.001, 0.043, 0.004, and <0.001, respectively, Table 4).

**Table 4:** Comparative analysis of baseline and 1‐month periodontal parameters in sampling sites.

Clinical parameters	Test group		*p*_1_‐Value	Control group		*p*_1-_Value	*p*_2_‐Value
	Baseline	1st month		Baseline	1st month		
PDs (mm)							
Mean ± SD	8.55 ± (1.5)	7.15 ± (2)	**<0.001** *⁣* ^∗∗∗^	8.3 ± (1.5)	8.45 ± (1.8)	0.317	**<0.001** *⁣* ^∗∗∗^
Median (IQR)	8.5 (2.8)	6.5 (3.5)		8 (2)	8.5 (2.8)		
Min–max	7–11	5–11		6–11	6–11		
CALs (mm)							
Mean ± SD	9.15 ± (2.8)	8.15 ± (2.5)	**0.009** *⁣* ^∗∗^	9.55 ± (1.9)	9.7 ± (2.2)	0.317	**<0.001** *⁣* ^∗∗∗^
Median (IQR)	9 (3.8)	7.5 (4.8)		9 (3)	10 (3)		
Min–max	1–14	5–14		7–13	6–14		
Pıs							
Mean ± SD	1.8 ± (0.5)	0.2 ± (0.5)	**<0.001** *⁣* ^∗∗∗^	1.75 ± (0.6)	0.85 ± (1)	**0.005** *⁣* ^∗∗^	**0.043** *⁣* ^∗^
Median (IQR)	2 (0.8)	0 (0)		2 (1)	0.5 (2)		
Min–max	1–3	0–2		1–3	0–3		
Gıs							
Mean ± SD	2.05 ± (0.2)	1.40 ± (0.5)	**<0.001** *⁣* ^∗∗∗^	2	1.8 ± (0.4)	**0.046** *⁣* ^∗^	**0.004** *⁣* ^∗∗^
Median (IQR)	2 (0)	1 (1)		2 (0)	2 (0)		
Min–max	2–3	1–2		2–2	1–2		
BOPs (%)							
Mean ± SD	100	35 ± (48.9)	**<0.001** *⁣* ^∗∗∗^	100	100	1.000	**<0.001** *⁣* ^∗∗∗^
Median (IQR)	100 (0)	0 (100)		100 (0)	100 (0)		
Min–max	100–100	0–100		100–100	100–100		

*Note:* p_1_: Comparison within groups (baseline and 1st month) (Wilcoxon test). p_2_: Comparison between groups (test and control) based on the changes in clinical parameters between two‐time intervals (Mann–Whitney *U* test).

Abbreviations: BOPs, bleeding on probing in sampling sites; CALs, clinical attachment level in sampling sites; GIs, gingival index in sampling sites; IQR, interquartile range; PDs, probing depth in sampling sites; PIs, plaque index in sampling sites; SD, standard deviation.

*⁣* ^∗^ *p*  < 0.05.

*⁣* ^∗∗^ *p*  < 0.01.

*⁣* ^∗∗∗^ *p*  < 0.001.

Table 5 presents the linear relationships between changes in clinical parameters and the fold changes in *Pg*, *Td*, and *Tf*. Analysis could not be conducted for BOP at the sampling sites due to a constant value (100%) across all samples in the control group. No significant relationship was found between changes in periodontal parameters and the fold changes of *Pg*, *Td*, and *Tf* in either group (*p* > 0.05). However, a strong positive correlation was observed between the fold changes of *Td* and *Tf* in the control group (*r* = 0.713, *p*  < 0.001), while a moderate positive correlation was found between the fold changes of *Pg* and those of *Td* and *Tf* in the test group (*r* = 0,651, *p* = 0.002; *r* = 0.583, *p* = 0.007, respectively, Table 5).

**Table 5:** Association between fold changes in bacterial counts and changes in periodontal parameters of sampling sites.

Parameters		*P. gingivalis* (2^−∆∆Ct^)	*T. denticola* (2^−∆∆Ct^)	*T. forsythia* (2^−∆∆Ct^)
Control group				
*Δ*PDs	r	−0.147	−0.408	−0.161
	p	0.535	0.074	0.498
*Δ*CALs	r	−0.147	−0.408	−0.161
	p	0.535	0.074	0.498
*Δ*PIs	r	0.242	0.254	−0.082
	p	0.305	0.280	0.732
*Δ*GIs	r	0.195	−0.282	−0.130
	p	0.410	0.229	0.585
*P. gingivalis* (2^−∆∆Ct^)	r	1.000	0.382	0.329
	p	—	0.097	0.156
*T. denticola* (2^−∆∆Ct^)	r	0.382	1.000	**0.713**
	p	0.097	—	**<0.001*⁣* ^∗∗^**
*T. forsythia* (2^−∆∆Ct^)	r	0.329	**0.713**	1.000
	p	0.156	**<0.001*⁣* ^∗∗^**	—
Test group				
*Δ*PDs	r	0.272	0.302	0.225
	p	0.245	0.195	0.341
*Δ*CALs	r	0.071	0.323	0.087
	p	0.766	0.164	0.716
*Δ*PIs	r	0.015	−0.044	0.118
	p	0.949	0.854	0.620
*Δ*GIs	r	−0.264	−0.282	0.009
	p	0.261	0.229	0.970
*Δ*BOPs	r	0.082	−0.082	0.100
	p	0.732	0.732	0.675
*P. gingivalis* (2^−∆∆Ct^)	r	1.000	**0.651**	**0.583**
	p	—	**0.002*⁣* ^∗^**	**0.007*⁣* ^∗^**
*T. denticola* (2^−∆∆Ct^)	r	**0.651**	1.000	0.415
	p	**0.002*⁣* ^∗^**	—	0.069
*T. forsythia* (2^−∆∆Ct^)	r	**0.583**	0.415	1.000
	p	**0.007*⁣* ^∗^**	0.069	—

*Note:* Bold values indicate statistically significant correlations. r, Spearman’s rho correlation coefficient. C_t_, threshold cycle values for the target pathogen; 2^−∆∆Ct^, the fold change in quantities of the target pathogen in the first month compared to the baseline.

Abbreviations: BOPs, bleeding on probing in sampling sites; CALs, clinical attachment level in sampling sites; GIs, gingival index in sampling sites; PDs, probing depth in sampling sites; PIs, plaque index in sampling sites.

*⁣* ^∗^ *p*  < 0.05.

*⁣* ^∗∗^ *p*  < 0.001.

## 4. Discussion

The objective of the present study was to assess the short‐term effects of AFS on the levels of red‐complex periodontopathogens and periodontal parameters, with a specific focus on Stage III, Grade B periodontitis patients with deep residual pockets (PD ≥ 6 mm). Stage III was prioritized due to its higher prevalence compared to Stage IV, where severe tooth loss limits sampling [22]. In line with our methodology, the current guideline [7] recommends surgical treatment for stage III patients with deep pockets (PD ≥ 6 mm) after initial therapy, aligning with evidence identifying this depth as the threshold for surgical intervention [23]. According to the clinical guidelines [7], AFS is recommended in the presence of deep residual pockets (PPD ≥ 6 mm) in patients with Stage III periodontitis after the first and second steps of periodontal therapy, while repeated SI is suggested for moderately deep residual pockets (4–5 mm). In the present study, the test quadrants exhibited deep residual pockets; therefore, AFS was directly performed. Repeated instrumentation was not applied, as additional debridement without adjunctive therapies was unlikely to provide further benefit, and adjunctive agents were intentionally avoided to prevent any influence on the microbiological outcomes. However, it should be acknowledged that the absence of retreatment in the control quadrants after initial SI represents a methodological choice to isolate the surgical effect and does not fully reflect routine clinical practice in which residual pockets would typically receive further instrumentation within this time frame. This design may therefore have accentuated between‐group differences by allowing greater bacterial recolonization on the control side.

Unlike previous PCR‐based methods, qPCR allows for the determination of the nucleotide sequence of the target gene, enabling the detection, quantification, and characterization of pathogens [24]. The use of TaqMan probes in qPCR allowed specific bacterial probes to generate fluorescent signals with a broad dynamic range, making this a particularly reliable method for quantifying *Pg* [25]. Furthermore, Coffey et al. [19] suggested that the TaqMan assay provides a rapid and efficient tool for profiling and quantifying periodontopathogens. Another similar study found that TaqMan assays are easier to interpret than SYBR Green due to reduced background noise and improved sensitivity [26]. Consequently, in our study, TaqMan probe was employed to achieve high‐sensitivity quantification of red‐complex species.

Subgingival samples obtained 1 month post‐AFS showed that surgical treatment significantly reduced periodontopathogen quantities compared to nonsurgically treated quadrants. In the test group, qPCR revealed a 12.21‐fold reduction in *Pg*, a 12.08‐fold reduction in *Td*, and an 18.94‐fold reduction in *Tf* relative to baseline levels. Correspondingly, in a randomized controlled trial, Martins et al. [27] demonstrated a significant reduction in red‐complex bacterial levels, particularly *Td*, following surgical periodontal treatment. In a comparable finding, Gokhale et al. [28] reported a statistically significant reduction in colony‐forming units of obligate anaerobes following AFS. Other similar studies also indicated an immediate reduction in the levels of red‐complex species following surgery [27, 28]. Consistent with the present study, Levy et al. [30] stated that the mean counts of 19 out of 40 subgingival species were significantly reduced following apically repositioned flap surgery, with significantly greater reductions observed in members of the red‐complex after surgical periodontal treatment compared to the NSPT group. Nevertheless, Paul et al. [31] stated that NSPT with antimicrobial agents is as effective as AFS in suppressing pathogenic microflora in moderate and deep pockets, though they emphasized the necessity of access surgery for unresponsive cases or hard‐to‐reach sites. The findings of the current study indicate that AFS largely reduces red‐complex pathogens and supports the development of a less pathogenic environment.

The control group in the present study exhibited a significant increase in the quantities of subgingival periodontopathogens. Similar studies suggest that although the levels of red‐complex periodontopathogens initially decrease following SI, biofilm pathogenicity may be regained within approximately 4 weeks [32, 33]. Sbordone et al. [14] reported that, by the third week after SI, subgingival microflora began reverting to its pretreatment state, with a subsequent increase in periodontopathogens. Another study showed that bacterial load typically returns to baseline within 2–6 months after debridement [34]. In this study, sampling in the control group was conducted at two time points: at enrollment (1 month post‐SI) and 1 month after AFS (approximately 2 to 3 months post‐SI). The notable increase in periodontopathogens between these two points aligns with aforementioned findings. However, because the control quadrants did not undergo retreatment during the follow‐up period, recolonization may have progressed more rapidly than under standard maintenance conditions, and thus the observed between‐group microbiological differences should be interpreted with caution. Nevertheless, Levy et al. [30] documented comparable reductions in pathogen levels at both surgical and nonsurgical sites 3 months postsurgery, suggesting that differences observed in the early healing phase may diminish over longer follow‐up periods.

The microbiological findings observed in the present study may be corroborated by the clinical results. Although significant reductions in all periodontal parameters were observed in both groups, AFS resulted in greater PD reduction and CAL gain in the test group. In this group, a 1.45‐mm greater reduction in PD was observed, while a 0.45 mm greater gain in CAL was achieved. Additionally, the test group showed significantly greater improvements in both PI and GI values, with BOP reducing from 90% to 28% at surgical sites. In accordance, most randomized clinical trials have documented that surgical treatment in patients with severe periodontitis leads to reductions in median values of PD, CAL, PI, GI, and BOP [35–37]. Consistently, a meta‐analysis demonstrated a significant reduction in PD and CAL following periodontal surgery [24]. In a study conducted by Sanz‐Sanchez et al. [36], it was concluded that the AFS procedure applied to deep pockets (≥6 mm) resulted in greater PD reduction compared to SI; however, no difference was detected in CAL gain. In two other split‐mouth studies, which align with our findings, a significant decrease in PD and BOP values and a significant increase in mean CAL gain were observed at both sites, with a more pronounced improvement at the surgically treated sites [29, 30]. Moreover, Heitz‐Mayfield et al. [23] noted that the reduction in PD following periodontal surgery in pockets ≥6 mm was 0.6 mm greater than in NSPT sites, with an additional 0.2 mm of attachment gain. Although reductions in bacterial load and improvements in clinical parameters occurred concurrently, our analyses did not reveal a direct statistical association between these changes. Therefore, the coexistence of these trends should not be interpreted as evidence of causality but rather viewed as parallel phenomena within the short‐term healing process.

The findings of the present study suggest that AFS reduces clinical inflammation by removing biofilm and infected tissue, with pocket reduction likely due to gingival shrinkage from decreased inflammation and partial new attachment. However, the BOP percentage remained above 10%, a threshold set by the 2017 workshop [10] as indicative of periodontal health, possibly due to patients avoiding brushing the surgical site immediately after surgery. A limited degree of clinical improvement was also evident in the control quadrants, most likely due to the effects of initial SI, reinforcement of oral hygiene, and the patient’s innate healing capacity. These improvements, however, remained modest compared with the surgical quadrants. At control sites, PD reduction and CAL gain were minimal and BOP frequently persisted, whereas surgically treated sites demonstrated substantially greater PD and BOP reductions together with significant CAL gain. Overall, these observations may support the concept that while nonsurgical therapy may provide some benefit, deep residual pockets respond more predictably and favorably to surgical access procedures.

No significant correlation was found between changes in periodontopathogens and periodontal parameters in either group. However, in the test group, a positive correlation was observed between the fold change in *Pg* and both *Tf* and *Td*, while the control group showed a positive correlation between *Td* and *Tf*. Red‐complex species are known to synergistically contribute to the progression of periodontitis, particularly through cross‐feeding mechanisms [38]. Our findings align with those of Gmür et al. [39], who identified a strong relationship between *Tf* and *Pg*, with the latter being undetectable in the absence of the former. Similarly, Byrne et al. [40] emphasized that *Pg* is rarely found in subgingival biofilm without *Td* and *Tf*. Additionally, a study suggests that *Tf* may colonize the plaque before *Pg* and *Td*, thereby facilitating their subsequent presence [41]. Nevertheless, as no significant correlations were detected between microbial shifts and clinical improvements in the present study, these outcomes should be regarded as occurring in parallel rather than exerting a direct causal influence on one another.

The main limitation of this study is its short‐term assessment of the microbiological effects of AFS, as subgingival plaque samples were collected only at baseline and 4 weeks postsurgery. Although some studies have evaluated earlier or slightly extended time points [13, 42], most clinical trials recommend longer follow‐up periods, typically ranging from 3 months to 1 year [30, 35, 43], to more fully capture the microbial and clinical dynamics of healing. The relatively small sample size and the possible influence of obtaining microbiological samples 1 month after surgery on periodontal healing also represent limitations. Furthermore, because the control quadrants did not receive retreatment during the observation period—a methodological decision intended to avoid confounding—bacterial recolonization may have progressed more rapidly than under standard maintenance care, potentially exaggerating the differences between groups. Finally, the considerable variability and wide dispersion observed in the fold‐change values reflect inherent biological heterogeneity among sites, which may have influenced the magnitude and consistency of treatment responses. Further multicenter, long‐term, large‐sample‐size, randomized controlled clinical studies are needed to fully assess the impact of AFS on periodontal tissues and subgingival flora.

## 5. Conclusion

Following AFS, a significant reduction in the levels of red‐complex periodontopathogens was observed, accompanied by more substantial improvements in clinical parameters at the surgical sites. In conclusion, surgical treatment seems to foster an environment more conducive to periodontal health by effectively reducing pathogenic species in the subgingival biofilm and improving clinical parameters, thus supporting better overall periodontal outcomes.

## Ethics Statement

This study was performed in line with the principles of the Declaration of Helsinki. The protocol of this clinical research was approved with the EKAEK 05/07 code by the Clinical Research Ethics Committee of Trakya University.

## Consent

Each participant was informed prior to the study, and signed informed consent forms were obtained.

## Disclosure

All authors gave final approval and agreed to be accountable for all aspects of the work, ensuring its integrity and accuracy.

## Conflicts of Interest

The authors declare no conflicts of interest.

## Author Contributions

contributed to the design, funding acquisition, medical ethics approval, surgery, and critical revision of the manuscript. Ece Açıkgöz‐Alparslan contributed to the conception and design, data collection, data interpretation, data analysis, and drafting of the original manuscript.

## Funding

This study was funded by the Trakya University Scientific Research Projects (Project Number: 2021/80).

## Supporting Information

Additional supporting information can be found online in the Supporting Information section.

## Supporting information


**Supporting Information** CONSORT guideline: The CONSORT checklist, used to standardize clinical studies and ensure transparency in their evaluations, has been prepared for the review process. The completed checklist is included in the supporting files upon submission.

## Data Availability

The data that support the findings of this study are available on request from the corresponding author. The data are not publicly available due to privacy or ethical restrictions.
